# The Cultivation of Bt Corn Producing Cry1Ac Toxins Does Not Adversely Affect Non-Target Arthropods

**DOI:** 10.1371/journal.pone.0114228

**Published:** 2014-12-01

**Authors:** Yanyan Guo, Yanjie Feng, Yang Ge, Guillaume Tetreau, Xiaowen Chen, Xuehui Dong, Wangpeng Shi

**Affiliations:** 1 Department of Entomology, China Agricultural University, Beijing, China; 2 Department of Entomology, Cornell University, New York State Agricultural Experiment Station, Geneva, New York, 14456, United States of America; 3 Department of Agriculture Science, China Agricultural University, Beijing, China; French National Institute for Agricultural Research (INRA), France

## Abstract

Transgenic corn producing Cry1Ac toxins from *Bacillus thuringiensis* (Bt) provides effective control of Asian corn borer, *Ostrinia furnacalis* (Guenée), and thus reduces insecticide applications. However, whether Bt corn exerts undesirable effects on non-target arthropods (NTAs) is still controversial. We conducted a 2-yr study in Shangzhuang Agricultural Experiment Station to assess the potential impact of Bt corn on field population density, biodiversity, community composition and structure of NTAs. On each sampling date, the total abundance, Shannon's diversity index, Pielou's evenness index and Simpson's diversity index were not significantly affected by Bt corn as compared to non-Bt corn. The “sampling dates” had a significant effect on these indices, but no clear tendencies related to “Bt corn” or “sampling dates X corn variety” interaction were recorded. Principal response curve analysis of variance indicated that Bt corn did not alter the distribution of NTAs communities. Bray-Curtis dissimilarity and distance analysis showed that Cry1Ac toxin exposure did not increase community dissimilarities between Bt and non-Bt corn plots and that the evolution of non-target arthropod community was similar on the two corn varieties. The cultivation of Bt corn failed to show any detrimental evidence on the density of non-target herbivores, predators and parasitoids. The composition of herbivores, predators and parasitoids was identical in Bt and non-Bt corn plots. Taken together, results from the present work support that Bt corn producing Cry1Ac toxins does not adversely affect NTAs.

## Introduction

Genetically modified (GM) crops have been planted for two decades since the first commercialized GM crop was released in 1994 [1]. The global cultivated surface of GM crops has increased from 1.7 million ha in 1996 to 175.2 million ha in 2013 [2]. In 2013, 18 million farmers benefited from planting GM crops in more than 30 countries worldwide [2]. The most famous and widespread GM crops are those producing *Bacillus thuringiensis* (Bt) toxins, which represent the most environmentally-safe alternative to chemical insecticides for pest control in agriculture [3], [4]. In China, largely owing to the cultivation of Bt cotton against cotton bollworm, *Helicoverpa armigera* (Hübner) (Lepidoptera: Noctuidae), the use of insecticides decreased greatly [5]–[7]. The cultivation of Bt corn can even decrease insecticide applications in agricultural fields by more than 50% [8].

The Asian corn borer (ACB), *Ostrinia furnacalis* (Guenée) (Lepidoptera: Pyralidae), is the most damaging lepidopteran pest of corn in China, with an estimated annual loss ranging from 6 to 9 million tons of corn [9]. The primary methods for controlling ACB include insecticide applications, alternative cultural practices (*e.g.*, crop rotation, tillage practices and mineral nutrition), mating disruption technique and parasitoid conservation and artificial releases [10]–[12]. Unfortunately, these approaches suffer from severe limitations: the efficacy of insecticide applications is often limited because most ACB larvae develop inside the stalk, limiting their exposure to the insecticides [13], [14] and they easily develop resistance to insecticides [15], [16], while the other control methods cited above may be very costly or too laborious for routine use. A safe and efficient alternative to these approaches would be the use of Bt corn since it may provide effective control of corn borers [17]. A recent work emphasized the effectiveness of Bt corn against ACB, reducing leaf injury by 84% and borer tunnels by 99% [18]. A new corn variety producing Cry1Ac toxins, which was mainly targeting ACB, has been developed in recent years. Although commercialization of Bt corn has not been allowed in China yet, field trials have been approved to monitor its impact on target pests and non-target organisms [19]. In the foreseeable future, they may be commercially available in China. Before commercialization, field studies are necessary to monitor the environmental risks of Bt corn. Therefore, we conducted this study to assess the risks of this new corn variety on non-target arthropods (NTAs).

NTAs include all species other than those that pest management actions are intended to suppress [20]. In corn ecosystems, NTAs provide very important ecological functions such as biological control, regulation of arthropod pest populations, organism decomposition, recycling of organic matter and pollination [21], [22]. The use of Bt corn may have direct (*e.g.*, through host/prey ingestion) [3] or indirect (*e.g.*, through food web interactions, scale of adoption) [22] effects on NTAs that may interfere with these functions [21]. In general, it is necessary to conduct field studies when the main potential effects of Bt crops are caused by complex interactions that cannot be evaluated in simple laboratory conditions [21]. In the past decades, field studies have been conducted in many countries, where the authors have assessed the impact of Bt corn on NTAs [23], [24]. Most of the studies reported no harmful impact of Bt corn on NTAs [25]–[27]. Only few studies reported that Bt corn affected NTAs [28]–[30], but the involvement of Bt traits in these studies was not proved because the results were governed by many interacting and uncontrollable factors [31]. In any ecosystem, there is a high number of NTAs species that may be exposed to GM plants. However, not each of these species can be tested. A representative subset of NTAs species (referred to as “focal species”) should be selected for consideration on the risk assessment of GM plants [22]. European Food Safety Authority (EFSA) provided guidelines for selecting focal species for such studies [22]. They suggested to choose species that can be easily tested under laboratory conditions and that are more likely to be available in sufficient number in the field to give statistically meaningful results [32]–[34]. They provided some examples of focal species of non-target organisms that can be used to monitor the impact of Bt plants [22], which include non-target herbivores (*e.g.*, aphids, leafhoppers, thrips, leaf beetles), non-target natural enemies (*e.g.*, flower bugs, lacewings, ladybird beetles, parasitoids), pollinators (*e.g.*, social bees, hover flies), and decomposers (*e.g.*, dipteran larvae, springtails). Meanwhile, they pointed out that additional species should also be included, such as species of economic, aesthetic or cultural value, or species of conservational importance that are threatened or endangered [22]. In this study, we chose non-target herbivores and natural enemies as focal species due to their abundance in the field and ecological functions in agro-ecosystems.

The objective of this study was to evaluate the population density, biodiversity, community composition and structure of NTAs on Bt corn producing Cry1Ac toxins and the corresponding non-transformed near-isogenic corn in the field, thus providing a theoretical basis for environmental risk assessment of transgenic plants. With this aim, the abundance of NTAs in Bt and non-Bt corn plots was recorded. Bray-Curtis dissimilarity has been widely used for multivariate analysis of community data [35], but no data was available for the dissimilarities of NTAs communities between Bt and non-Bt corn plots. Therefore, we analyzed the Bray-Curtis dissimilarities between Bt and non-Bt corn plots to provide a method for monitoring the impact of Bt corn on NTAs.

## Materials and Methods

### Bt and non-Bt corn

The Bt corn (Bt 799) and the corresponding non-transformed near isoline (Zheng 58, non-Bt) used in this field experiment were provided by China National Corn Center. The transgenic corn contains a gene encoding *Bacillus thuringiensis* Cry1Ac toxin, toxic for Asian corn borer (ACB). Cry1Ac levels in Bt corn leaves ranged from 310.4 ng.g^−1^ to 597.67 ng.g^−1^ (fresh weight) (Xiaowen Chen, unpublished data). Using the same method as described by He [9], we found that leaf feeding rating by ACB in this Bt corn was 1.09 (low infection) while that in the non-Bt corn was 6.81 (high infection), indicating that Bt corn producing Cry1Ac toxins can control ACB effectively.

### Experimental design

The study was conducted in the field of Shangzhuang Agricultural Experiment Station (Altitude, 47 m; 116°17′52.84″E; 39°57′52.84″N), Haidian District,Beijing, China, in 2012 and 2013. The field was newly explored in 2011, where no crops, including Bt crops, were planted here before. Bt and non-Bt corn plots were arranged in a randomized block design with 3 replications, respectively. Each plot measured 10 m by 15 m and contained 16 rows with 60 cm between them and 25 cm between individual plants. 3 m bare borders were established to serve as isolation among plots. A 3 m strip border around the perimeter was planted with non-Bt corn (Fig. S1). No plants were grown in the study field until the experiment started. No herbicide or insecticide were applied before or during the study period. In 2012 and 2013, corn was planted on June 10 and May 7, respectively.

### Field sampling and species identification

In each plot, 100 corn plants were sampled following an “X” pattern that covered the whole plot (Fig. S1). In order to avoid edge effects, the sampling began at approximately 2 m into the plot [36]. The abundance of all arthropods for each plant, including stems and both sides of the leaves, were surveyed carefully by visual sampling. Sampling was conducted early in the morning when arthropods were less active [25]. Unknown species collected were preserved in 75% alcohol for further identification in the laboratory. Sampling dates between 2012 and 2013 are shown in Table S1. The arthropods collected were identified at the species level, whenever possible. When samples were too degraded and/or when it was hard to distinguish the morphological criteria, identification was performed at the family level.

### Statistical analysis

NTAs total abundances (N) were log(x) transformed prior to analysis. Diversity indices such as Shannon's diversity index (H), Pielou's evenness index (J) and Simpson's diversity index (D) allow a comparison of the community structures between different treatments in the fields [37]. All these indices are sensitive to the abundance of the most common and dominant species in a community [38], [39]. H, J and D were analyzed [40] using linear mixed models (*lmer* function of R package *lme4*), with corn variety (Bt or non-Bt) and time (sampling date) as fixed factors. On each sampling date, mean values of N, H, J and D were compared using a one-way ANOVA to detect significant differences between Bt and non-Bt corn plots.

Principal response curve (PRC) is a particular ordination method of RDA (redundancy analysis) firstly introduced by van den Brink and ter Braak [41], which is especially suitable for the evaluation of ecosystem experiments [42]. It is a multivariate technique allowing to assess the structure of species community [43], making it suitable to investigate the impact of Bt corn on NTAs and their changes over time. It can also be used for future monitoring studies [44]. Potential changes in the structure of the NTAs communities due to Cry1Ac toxin exposure were analyzed using the PRC method [45]–[47]. In most published papers, the PRC was performed with CANOCO [23], [45], [48]–[50] but additional calculations were necessary to obtain the canonical coefficients (C*dt*) values. In the present study, we used the RDA model (*prc* function of R package *vegan*) instead of CANOCO, which is much easier to perform and provide all the *Cdt* values directly.

Bray-Curtis index is a measure of dissimilarity between two samples and ranges from 0 (similar) to 1 (dissimilar) [51], [52]. Temporal changes in the dissimilarities of NTAs communities between Bt and non-Bt corn plots were performed using Bray-Curtis index calculation. The first analysis was performed between Bt and non-Bt corn plots. On each sampling date, Bray-Curtis distance was calculated for all the pairs of the two corn plots. The mean values and standard errors were then computed. The second analysis was performed within each corn plot [53]. On each sampling date, the mean abundance of each taxonomic group in Bt and non-Bt corn plots was calculated. Mean abundance values were then calculated to get the Bray-Curtis distance between sampling dates for a given treatment. These values were linearly regressed against the time-lag values. The slopes of the regression lines obtained from the two treatments were compared by covariance analysis (ANOVA).

The arthropod community in corn plots was classified into three guilds based on nutritional relationships, *i.e.,* herbivores, predators and parasitoids, referring to Heong et al. [54] and Zhang et al. [55]. For each corn variety and date, the density of the three guilds was log (x+1) transformed prior to analysis and then analyzed using one-way ANOVA.

The proportion of herbivores, predators and parasitoids in each treatment is defined by the equation *P_i_* = N_i_/N, where N_i_ is the abundance of herbivores, predators or parasitoids and N is the overall total abundance in each treatment. The proportion of NTAs individuals in each guild is defined by the equation *P_i_* = N_i_/N, where N_i_ is the abundance of the i^th^ species and N is the total abundance of the guild in each treatment. *P_i_*<1% represents a rare group, 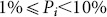 represents a common group and 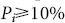 represents a dominant group [56]. Group proportion under 1% was gathered in “others”.

In this study, the means and *p* values of NTAs community descriptors and the density of herbivores, predators and parasitoids were calculated using SPSS for Windows, version 16.0 (SPSS Inc., IL, USA). All the other tests were performed using R for Windows, version 3.0.3 [57]. Statistical threshold was 0.05 for all the tests.

## Results

### Descriptors of the NTAs communities in Bt and non-Bt corn plots

**Figure 1:**
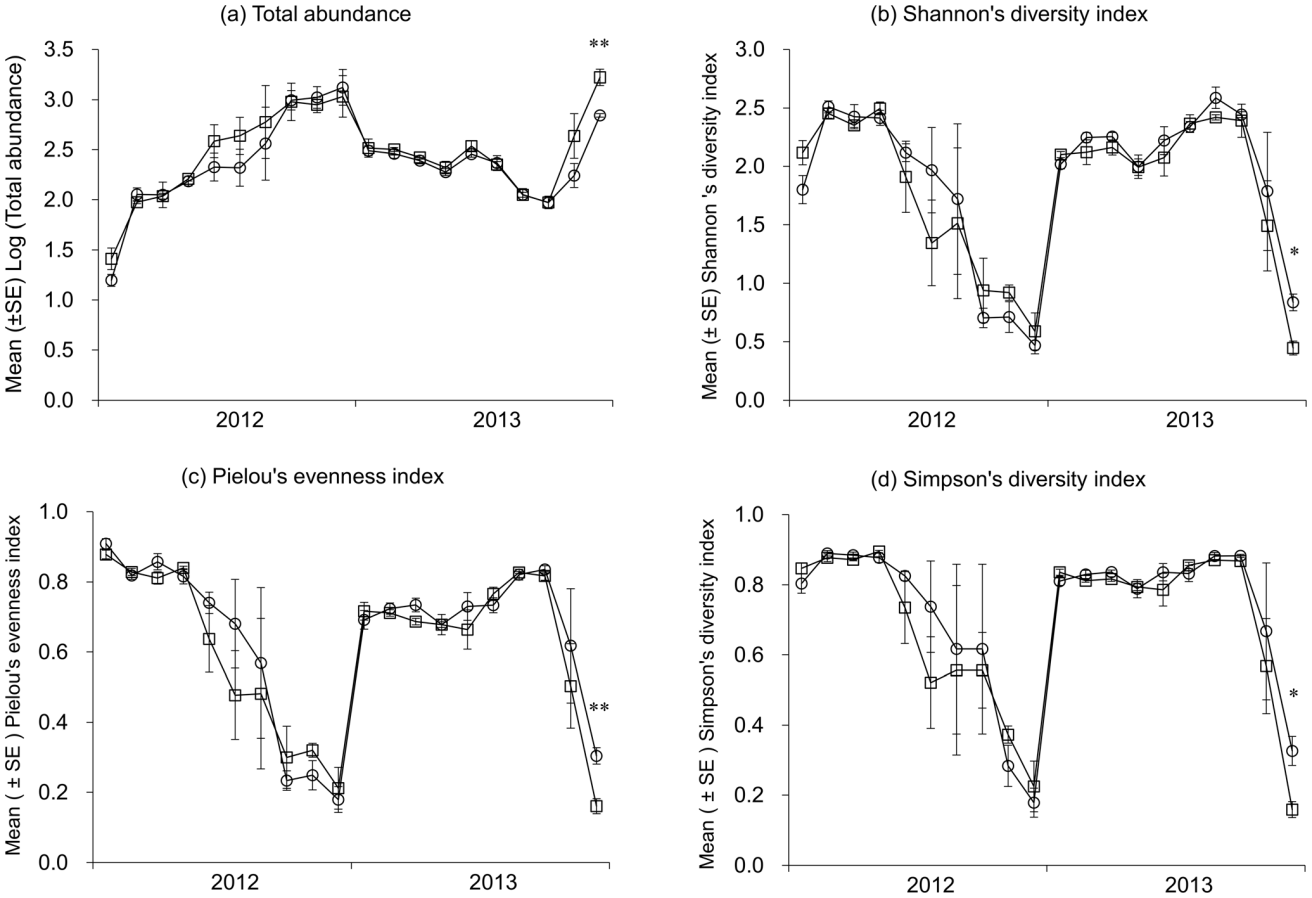
Changes in mean ± SE (n = 3) values of descriptors of the NTAs communities in Bt and non-Bt corn. (a) Total abundance; (b) Shannon's diversity index; (c) Pielou's evenness index; (d) Simpson's diversity index. Empty squares represent Bt corn and empty circles represent non-Bt corn. Statistically significant difference according to one-way ANOVA: *: 0.01<*p*≤0.05; **: 0.001<*p*≤0.01; ***: *p*≤0.001.

The variations for all descriptors of the NTAs community structure were monitored, and patterns were almost identical in the two treatments (Fig. 1). Of all the sampling dates, significant differences between Bt and non-Bt corn plots were only observed on August 27, 2013 for total abundance (N), Shannon's diversity index (H), Pielou's evenness index (J) and Simpson's diversity index (D) (*p* = 0.01, 0.013, 0.01, 0.024, respectively) (Fig. 1). During the two years, “corn variety” didn't have any significant effect on all the descriptors. In contrast, “time” had a highly significant effect on all the descriptors (Table 1). The effect of interaction between “time” and “variety” was not significant for all the descriptors (Table 1).

**Table 1:** Mean ± SE of the NTAs community descriptors in Bt and non-Bt corn plots during the whole study period (2012–2013).

NTAs community descriptors	Bt corn	Non-Bt corn	Time effect			Variety effect			Time×Variety		
			F	df	P	F	df	P	F	df	P
Total abundance (N)	481.47±102.91	399.57±91.29	9.13	19	<0.001	1.78	1	0.19	0.90	19	0.59
Shannon's diversity index (H)	1.81±0.15	1.88±0.15	21.26	19	<0.001	1.19	1	0.28	0.62	19	0.88
Pielou's evenness index (J)	0.62±0.05	0.65±0.05	23.65	19	<0.001	2.23	1	0.14	0.63	19	0.87
Simpson's diversity index (D)	0.69±0.05	0.72±0.05	14.32	19	<0.001	1.28	1	0.26	0.40	19	0.99

df, degrees of freedom; P, corresponding probability. All data was analyzed using linear mixed models.

### NTAs community response

**Figure 2:**
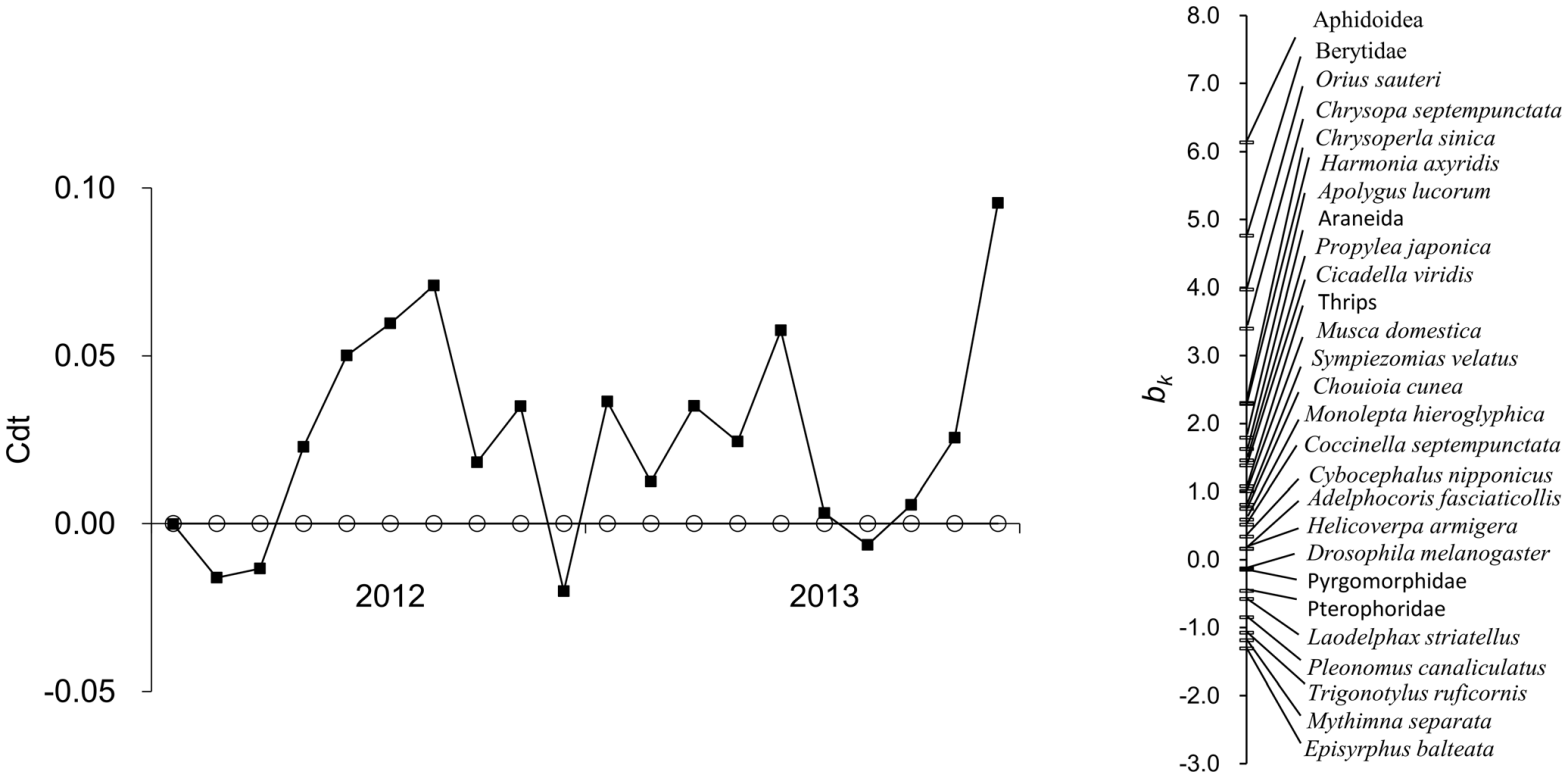
Principal response curve (PRC) resulting from the analysis of NTAs abundance dataset for the whole study period. The vertical axis represents the difference in community structure between Bt (filled squares) and non-Bt (empty circles) corn plots expressed as regression coefficients (*Cdt*) of the PRC model. The species weight (*b_k_*) can be regarded as the affinity of the taxon to the principal response. Only species with a weight less than -0.5 or greater than 0.5 are shown.

PRC analysis of NTAs abundance data revealed no significant differences (F = 1.27, *p* = 0.19) between Bt and non-Bt corn plots (Fig. 2). Of all the variance in the abundance data, 79.2% was attributed to the sampling dates, and only 4.1% was attributed to corn variety based on the first PRC. Tests for each sampling date indicated no difference between Bt and non-Bt corn plots. In Fig. 2, species weights between −0.5 and 0.5 were not shown because they are likely to show a weak response or a response that is unrelated to the principal response curve [45]. Analysis of the distribution of species weight (*b_k_*) confirmed that Aphidoidea, Berytidae, *Orius sauteri*, *Chrysopa septempunctata*, *Chrysoperla sinica*, *Harmonia axyridis*, *Apolygus lucorum*, Araneida, *Propylea japonica*, *Cicadella viridis*, thrips, *Musca domestica*, *Sympiezomias velatus*, *Chouioia cunea*, *Monolepta hieroglyphica*, *Cocconella septempunctata*, *Cybocephalus nipponicus*, *Adelphocoris fasciaticollis* and *Helicoverpa armigera* were more abundant in Bt corn plots than in non-Bt corn plots. In contrast, *Drosophila melanogaster*, Pyrgomorphidae, Pterophoridae, *Laodelphax striatellus*, *Pleonomus canaliculatus*, *Trigonotylus ruficornis*, *Mythimna separata* and *Episyrphus balteata* were less abundant in Bt corn plots, as compared to non-Bt corn plots.

### Bray-Curtis dissimilarity between Bt and non-Bt corn plots

**Figure 3:**
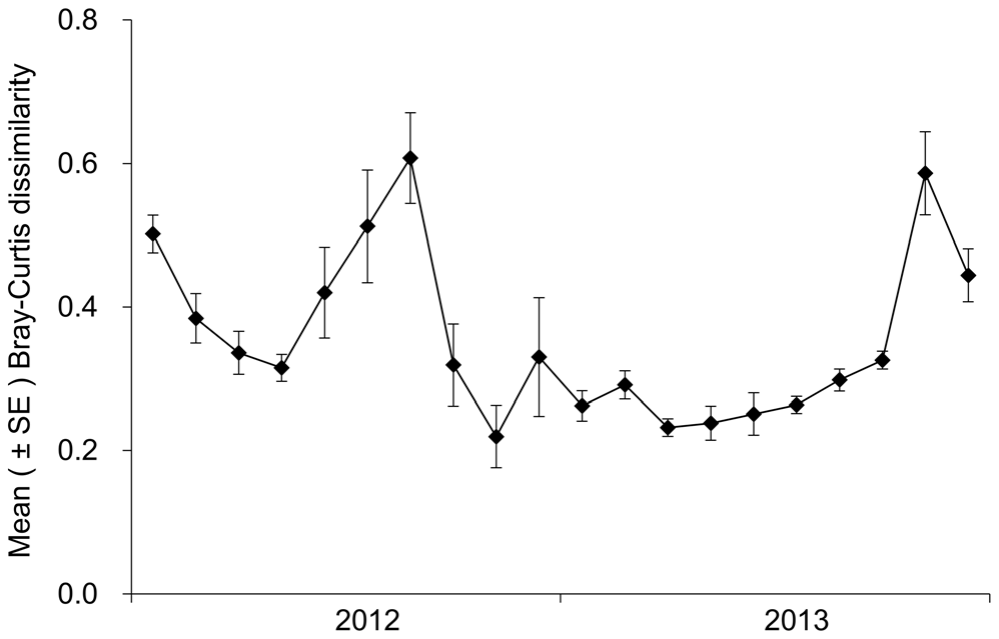
Changes in mean ± SE (n = 6) value of Bray-Curtis dissimilarity between Bt and non-Bt corn plots.

**Figure 4:**
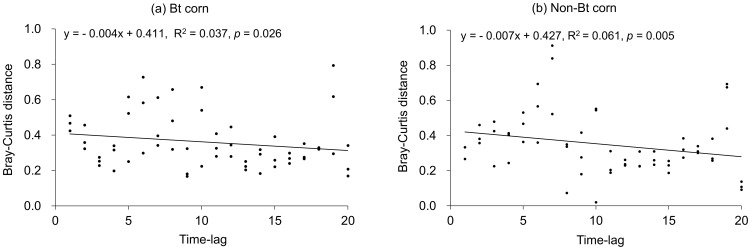
Time-lag analysis of NTAs community dynamics in Bt (a) and non-Bt (b) corn plots.

Mean values of Bray-Curtis dissimilarity between Bt and non-Bt corn plots fluctuated during the study period (Fig. 3). There was no significant relationship between Bray-Curtis dissimilarity values and elapsed time during the whole study period (Spearman r = −0.26, *p* = 0.26), indicating that Cry1Ac toxin exposure did not significantly altered NTAs community in Bt corn plots as compared to non-Bt corn plots. The relationship between Bray-Curtis dissimilarity and time lag among sampling dates was highly significant for both Bt (*p* = 0.026) and non-Bt (*p* = 0.005) corn plots (Fig. 4), suggesting that the structure of the NTAs communities evolved with time in the two corn plots. ANOVA analysis showed that the slopes of the relations were not significantly different (t = 2.28, *p* = 0.75), indicating that the rates of NTAs community changes were similar in the two corn plots.

### Density changes of herbivores, predators and parasitoids in Bt and non-Bt corn plots

**Figure 5:**
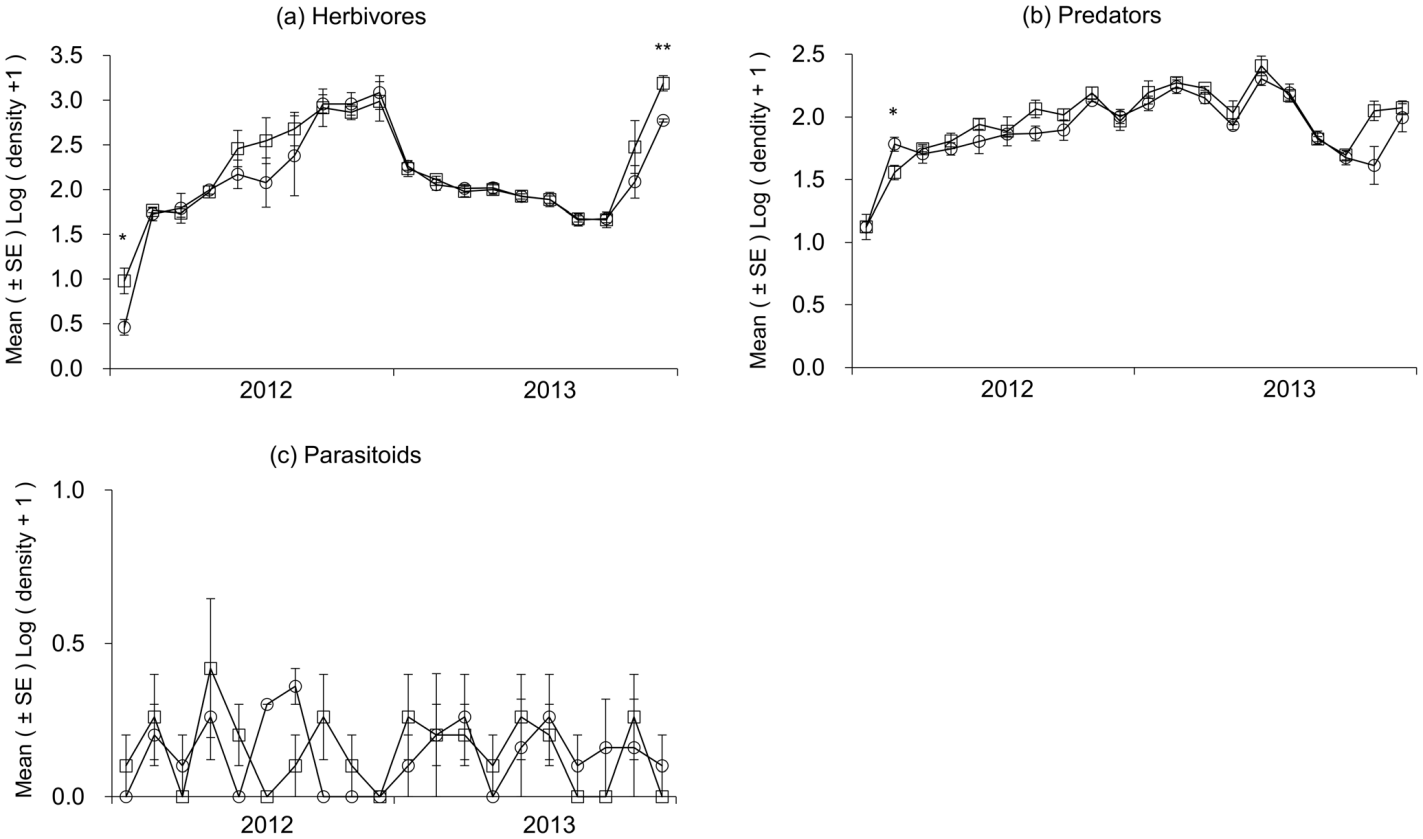
Changes in mean ± SE (n = 3) density of NTAs in Bt and non-Bt corn plots. (a) Herbivores; (b) Predators; (c) Parasitoids. Empty squares represent Bt corn and empty circles represent non-Bt corn. Statistically significant difference according to one-way ANOVA: *: 0.01<*p*≤0.05; **: 0.001<*p*≤0.01; ***: *p*≤0.001.

During the whole study period, the temporal dynamics of herbivores, predators and parasitoids density showed similar trends for Bt and non-Bt corn plots. For herbivores, significant differences between the two corn plots were only observed on July 16, 2012 (t = 3.10, df = 4, *p* = 0.036) and August 27, 2013 (t = 4.82, df = 4, *p* = 0.009) (Fig. 5a). On July 23, 2012, a significant difference was detected between the two corn plots for predators (t = −2.81, df = 4, *p* = 0.048) (Fig. 5b). No significant effect was observed for parasitoids density between Bt and non-Bt corn plots (Fig. 5c).

### Composition of NTAs communities in Bt and non-Bt corn plots

**Figure 6:**
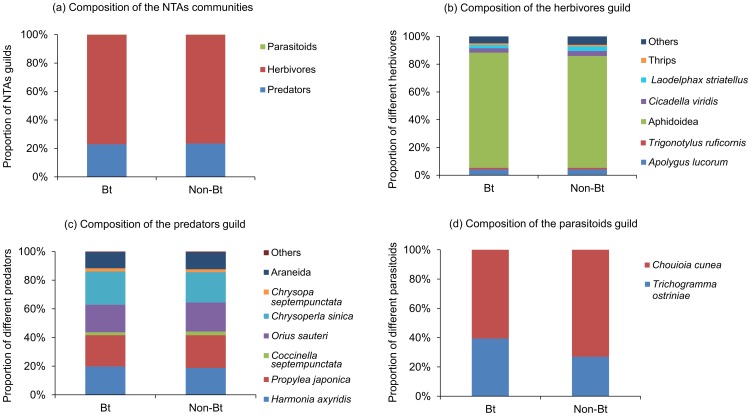
Composition of Bt and non-Bt corn NTAs communities. (a) NTAs communities; (b) Herbivores; (c) Predators; (d) Parasitoids.

Three guilds were identified in Bt and non-Bt corn plots during the study period. The results showed that the most abundant guilds in Bt and non-Bt corn plots were herbivores and predators. Parasitoids was a rare guild (Fig. 6a). Of herbivores, Aphidoidea was the most abundant. *Apolygus lucorum*, *T. ruficornis*, *C. viridis*, *L. striatellus* and thrips were the common groups (Fig. 6b). Of predators, *H. axyridis*, *P. japonica*, *O. sauteri*, *C. sinica* and Araneida were the most abundant groups. *Chrysopa septempunctata* represented the common groups (Fig. 6c). Of parasitoids, *T. ostriniae* and *C. cunea* were equally abundant (Fig. 6d). During the whole study period, the composition of NTAs communities was essentially uniform in Bt and non-Bt corn plots (Fig. 6).

## Discussion

The development of GM corn producing Cry toxins significantly reduced the use of insecticides in the environment [8], and thus may alleviate the risks of NTAs exposure to insecticides. With the large scale planting of transgenic Bt corn, an increasing number of scientists devoted to monitoring the environmental impact of Bt corn on NTAs [58]–[60]. In this study, the potential impact of Bt corn on NTAs was monitored during a 2-yr survey to assess the environmental risks associated with Bt corn cultivation. Ecological indices such as Shannon's diversity index, Pielou's evenness index and Simpson's diversity index are useful indicators of the disturbance of NTAs community condition [61]. If the cultivation of Bt corn disrupted the biological properties, the functional indices of NTAs would be significantly lower in Bt corn as compared to non-Bt corn. However, our results indicated that the total abundance of NTAs, Shannon's diversity index, Pielou's evenness index and Simpson's diversity index showed similar values in Bt and non-Bt corn plots in most cases. This suggested that Bt corn did not adversely affect the NTAs community structure. This finding was consistent with a previous 6-yr monitoring study showing that Bt corn did not affect NTAs community in German agricultural fields [62]. Similarly, a 2-yr study reported that environmental conditions (*e.g.*, heavy rainfall) or crop management practices had a greater impact on NTAs community than corn variety [23].

PRC analysis revealed no significant impact of Bt corn on NTAs distribution when compared to non-Bt corn. Among the 27 species/families monitored in this study, more than 70% of NTAs were more abundant in Bt corn plots than in non-Bt corn plots. It indicated that the presence of Bt toxin in the plant did not influence the population density of the assessed NTAs communities. This is consistent with previous work that found no effect of Bt corn producing Cry1Ab toxins on NTAs communities [63]. Also, community level analysis of the NTAs abundance performed in a 3-yr field study at four locations across the U.S. revealed no significant impact on community abundance in Bt corn fields when compared to non-Bt corn fields [64].

A large number of studies were conducted to assess the impact of Bt corn on NTAs. However, most of the previous studies focused on changes in NTAs abundance, resulting in the absence of data available for the dissimilarities of NTAs communities between Bt and non-Bt corn plots. To our knowledge, this is the first time the evolution dynamics of the NTAs communities are compared between Bt and non-Bt corn plots by measuring the Bray-Curtis dissimilarity index. Here we show that dissimilarities between Bt and non-Bt corn plots were small and not significant during the study period, indicating that the presence of Cry1Ac toxins in the corn did not induce any divergence in NTAs community structure. Furthermore, our analysis revealed some changes in the structure of the NTAs community over the 2 years, but the patterns of evolution were similar in both Bt and non-Bt corn plots.

To assess potential harm of Bt corn on NTAs, representative species of corn ecosystems need to be monitored when their relevant life stages are likely to be exposed to Bt toxins in the field [65]. In this study, we calculated the density of three representative guilds (herbivores, predators and parasitoids) based on their different nutritional relationships, which can result in a reliable result for the differences between Bt and non-Bt corn plots. Herbivores can be exposed to Bt toxins when consuming plant materials (*e.g.,* pollen, crop residues) [65], [66]. The main herbivores observed in this study were aphids, bugs, leafhoppers and thrips. Both laboratory and field studies showed that the density of these herbivores were not affected by Bt corn [62], [67]–[72]. Our results further supported the finding that Bt corn did not affect the density of herbivores. Predators can be in contact with Bt toxins in several ways: by feeding on plant materials or pollen, by feeding on target or non-target herbivores that have ingested Bt toxins, or via the environment (*i.e.,* the soil when Bt proteins persist and do not lose their toxicity after plants or insects have died) [73]. The biological functions provided by predators or natural enemies are mandatory for a good self-regulation of insect populations in agricultural ecosystems and they should not be harmed by the use of Bt corn [74]–[76]. Consequently, the evaluation of the impact of Bt corn on natural enemies should be addressed in the ecological risk assessment. The predators recorded in this study mainly included ladybird beetles, green lacewings, *Orius spp*. and spiders. Their densities were not affected by Bt corn, which was in agreement with previous findings in corn fields [77]–[82]. Some laboratory tritrophic studies further confirmed that predators had no preference between Bt and non-Bt corn fed prey [75], [83], [84]. Unlike predators, which can feed on different prey species, parasitoids usually complete their development on a single host individual [85]. Parasitoids can attack a variety of herbivores occurring in corn ecosystems [66], [86]. Thus, parasitoids could be affected by ingesting the Bt toxins present in host herbivores [87], [88]. Consistent with previous observations [89], parasitoids abundance was not adversely affected by Bt corn producing Cry1Ac toxins in our study. A meta-analysis of 20 field studies conducted in Spain from 1998 to 2010 to assess the risks of Bt corn on NTAs confirmed that the densities of herbivores, predators and parasitoids were not affected by Bt corn, which is consistent with our results [90].

The analysis of NTAs communities examined at the species/family level revealed that the composition of the abundant, common and rare guilds or species/families was similar in Bt and non-Bt corn plots. In field planted with Bt corn MON 88017 expressing Cry3Bb1 toxins, no effect was found on NTAs composition [65], which is in agreement with our findings.

No detrimental effect of Bt corn producing Cry1Ac toxins was observed on any NTAs community indices or on the abundance of NTAs. Moreover, PRC analysis suggested that cultivation of Bt corn did not alter the distribution of NTAs communities. Bray-Curtis analysis showed that NTAs communities evolved with a similar pattern in Bt and non-Bt corn plots. This study provides further evidence that the changes in the abundance and diversity of NTAs in corn plots are driven by time, and Cry1Ac toxin exposure only plays a negligible role, if any, in the evolution of these NTAs communities. Interactions between corn and NTAs occur over a wide range of time scales from hours to seasons and years and are mostly driven by temperature, rainfall or sunshine. Therefore, long-term and large-scale studies taking into account a large variety of environmental parameters, including the effect of potential insecticide treatments of non Bt crops, are still required to ensure a long term efficacy of GM crops with reduced impact on the environment and agricultural ecosystems [4].

## Supporting Information

Figure S1 Systematically randomized plot design with Bt corn and its non-transformed near isoline (Non-Bt).
(TIF)

Table S1 Dates of sampling in 2012 and 2013.
(DOCX)

File S1 Raw data.
(XLSX)
