# Supplementary figures and images for: The Cultivation of Bt Corn Producing Cry1Ac Toxins Does Not Adversely Affect Non-Target Arthropods
Source: PLoS One. 2014 Dec 1;9(12):e114228. doi: 10.1371/journal.pone.0114228 (PMC4250226; doi:10.1371/journal.pone.0114228)

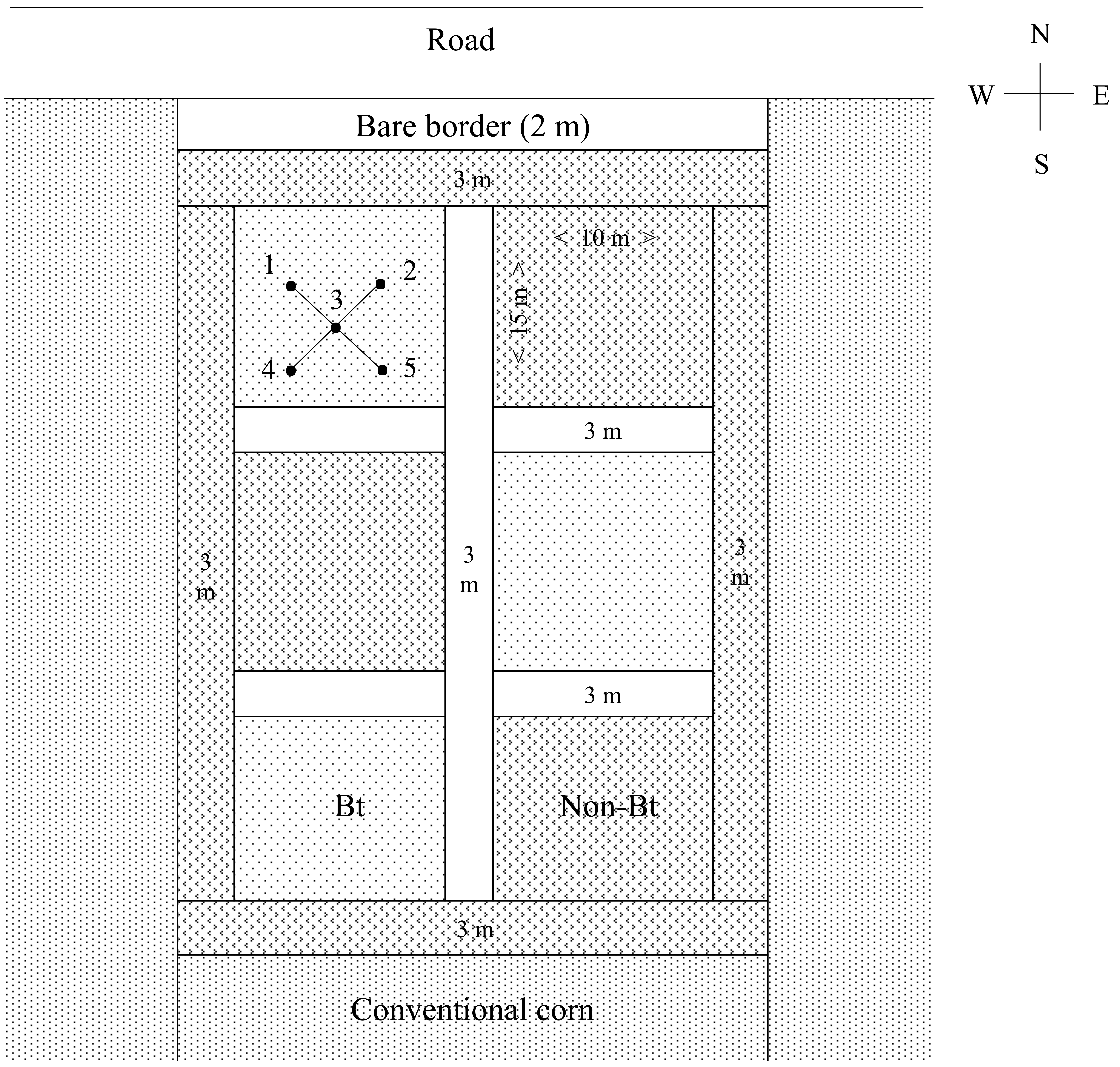

Supplement: Figure S1 — Systematically randomized plot design with Bt corn and its non-transformed near isoline (Non-Bt). (TIF) [file pone.0114228.s001.tif]
