# Supplementary material for: The Cultivation of Bt Corn Producing Cry1Ac Toxins Does Not Adversely Affect Non-Target Arthropods
Source: PLoS One. 2014 Dec 1;9(12):e114228. doi: 10.1371/journal.pone.0114228 (PMC4250226; doi:10.1371/journal.pone.0114228)
Supplement: Table S1 — Dates of sampling in 2012 and 2013. (DOCX) [file pone.0114228.s002.docx]

**Table S1.** Dates of sampling in 2012 and 2013.

| **Times** | **Sampling Date (mm/dd)** | |
| --- | --- | --- |
|  | **2012** | **2013** |
| 1 | 07/16 | 06/21 |
| 2 | 07/23 | 06/28 |
| 3 | 07/30 | 07/05 |
| 4 | 08/06 | 07/12 |
| 5 | 08/13 | 07/19 |
| 6 | 08/20 | 07/26 |
| 7 | 08/27 | 08/02 |
| 8 | 09/04 | 08/09 |
| 9 | 09/13 | 08/20 |
| 10 | 09/19 | 08/27 |
